# Time to Total Knee Arthroplasty (TKA) Post Intra-Articular Injection

**DOI:** 10.3390/jcm13133764

**Published:** 2024-06-27

**Authors:** Martin G. Gesheff, David A. Scalzitti, Sandeep S. Bains, Jeremy Dubin, Ronald E. Delanois

**Affiliations:** 1 Rubin Institute for Advanced Orthopedics, Sinai Hospital of Baltimore, LifeBridge Health, Baltimore, MD 21215, USA; mgesheff@lifebridgehealth.org (M.G.G.); sbains@lifebridgehealth.org (S.S.B.); jdubin2@lifebridgehealth.org (J.D.); 2 Health, Human Function, and Rehabilitation Sciences, George Washington University School of Medicine and Health Sciences, Washington, DC 20052, USA; scalzitt@gwu.edu

**Keywords:** total knee arthroplasty, intra-articular injection, corticosteroid, hyaluronic acid, platelet-rich plasma, time to total knee arthroplasty

## Abstract

**Background:** Disease-modifying treatments are not currently developed to target the underlying causes of knee osteoarthritis (KOA). Corticosteroids (CS), hyaluronic acid (HA), and platelet-rich plasma (PRP) intra-articular (IA) injections are commonly used for patients that do not respond to non-pharmacological treatments, oral nonsteroidal anti-inflammatory, or pain medications to address solely KOA symptoms. Utilizing TKA as an endpoint in the KOA disease progression provides a basis to determine efficacy of this treatment pathway. The primary objective is to evaluate a large national database to determine the time between first injection and total knee arthroplasty in patients solely administered intra-articular IA, CS, and HA. **Methods:** A retrospective query was performed on a national, all-payer claims database (PearlDiver, Colorado Springs, CO, USA), a composite of over 160 million Health Insurance Portability and Accountability Act compliant orthopedic records across all states and territories of the United States spanning 2016 to 2022. The database was queried to produce three distinct cohorts for analysis (PRP, HA, and CS). A 4:1 case match was conducted to compare cohorts receiving a subsequent TKA. Kaplan–Meier survival analysis analyzed the TKA-free survival of patients within each group at 6 months and 1 to 4 years. The log-rank test was performed for comparisons between survival cohorts. **Results:** The PRP cohort had a total population of 3240 patients, of which 71 (2.2%) received a subsequent TKA. The corticosteroid cohort had a total population of 1,382,572, of which 81,271 (5.9%) received a subsequent TKA. The HA cohort had a total population of 164,000, of which 13,044 (8.0%) received a subsequent TKA. Due to the low population within the PRP group, this group was excluded from comparison. The mean time to TKA from first injection in the HA group was 377.8 days, while in the corticosteroid group it was 370.0 days. The proportions of TKA-free survival for CS and HA when compared at 4 years post-injection was similar between groups (*p* = 0.05). **Discussion and Conclusion:** Patients that received only IA-corticosteroids or IA-hyaluronic acid had a similar length of time between the first injection and the total knee arthroplasty associated with the injected joint. This evidence provides information for clinicians and patients alike when contemplating these non-surgical injection modalities for KOA. The similarity observed between these treatments supports the need for future research to determine whether there is any potential for reduction in healthcare costs for KOA treatment prior to TKA.

## 1. Introduction

Osteoarthritis (OA) is a prevalent condition affecting approximately 7% of the global population and a major cause of years lived with disability [1]. This poses a significant challenge as effective disease-modifying treatments targeting its underlying causes are currently unavailable. Compounding this issue is the fact that knee osteoarthritis (KOA), a form of OA, is a progressive chronic disease often remaining asymptomatic in its early stages. While non-pharmacological interventions such as diet, education, physical therapy, lifestyle modifications, insoles, braces, or oral nonsteroidal anti-inflammatory drugs can be effective options for OA, corticosteroids (CS), hyaluronic acid (HA), and platelet-rich plasma (PRP) intra-articular (IA) injections are additional modalities that can be utilized to address symptoms in conjugation with non-pharmacological interventions [2,3].

Assessing the long-term efficacy of treatments for KOA is challenging due to the progressive nature of the disease. Clinical trials typically focus on primary endpoints related to pain and function, with cartilage structure assessment considered secondary or exploratory. Recently, there has been a push to consider total knee arthroplasty (TKA) as a primary endpoint in evaluating KOA treatments due to its direct clinical benefit to patients [4]. Delaying TKA can be advantageous, particularly in patients requiring optimization due to comorbidities or socioeconomic factors.

Despite the importance of IA injections in KOA management, few studies have investigated their impact on delaying TKA. Berkani et al. conducted a systematic review and meta-analysis, including over two million patients, which found that HA use was associated with a delay in TKA of approximately 9.8 months [2]. Concoff et al. reported that patients receiving multiple courses of HA experienced a median delay to TKA of 1.3 years compared to 0.38 years for those not receiving HA [5]. While IA corticosteroid injections have shown benefits in pain reduction and functional improvement, their effect on delaying TKA remains unclear [6,7]. Notably, long-term repeated use of CS injections has been associated with greater cartilage loss, potentially hastening the need for TKA [8].

There is a need to better understand the relationship between IA injections and the time until TKA in KOA patients. Utilizing TKA as an endpoint in disease progression provides a meaningful measure of treatment efficacy. Given the absence of true disease-modifying treatments for KOA, leveraging existing data to investigate the impact of IA injections on delaying TKA provided a major impetus for this study. Therefore, the primary objective of the current study is to evaluate a large national database to determine the time between the first injection and TKA in patients solely administered CS and HA.

## 2. Materials and Methods

A retrospective query was performed on a national, all-payer claims database (PearlDiver, Colorado Springs, CO, USA), a composite of over 160 million Health Insurance Portability and Accountability Act compliant records across all states and territories of the United States spanning 2016 to 2022. This subscription-based database is one of the largest aggregations of health care data with patient longitudinal data. The sources of this database include multiple private claims sources, Centers for Medicare and Medicaid Services inpatient, outpatient, and carrier standard analytical files, and Healthcare Cost and Utilization Project data. Patient cohorts and baseline patient demographic information were identified using International Classification of Diseases, tenth edition (ICD-10), procedural and diagnoses codes, as well as Current Procedural Terminology (CPT) codes. This study received institutional review board exemption as this study is retrospective and queried deidentified data.

### 2.1. Patient and Group Selection

The database was queried to produce three distinct cohorts for analysis and comparison (patients receiving only intra-articular injection of corticosteroids, hyaluronic acid, or platelet-rich plasma) utilizing International Classification of Disease (ICD) or Current Procedural Terminology (CPT) codes. A full list of the codes utilized to define knee osteoarthritis and each group for this study is provided in Appendix A. First, knee osteoarthritis was defined as the presence of either right or left laterality. Platelet-rich plasma patients were defined by querying PRP injection, using CPT code 0232T, and the presence of knee osteoarthritis. CPT code 0232T represents a procedure code for a PRP injection in any location, which necessitated this additional association coding. The remaining two groups were queried by associating the presence of knee osteoarthritis as previously defined, an injection (CPT 20610 or 20611), and the respective corticosteroid or hyaluronic CPT codes as defined in Appendix A. Only adult patients aged 18 and over were queried for these cohorts. Regardless of subsequent injections, patients’ first injection was the only injection captured to inform the primary outcome measure in this study. Case match cohorts (4:1) were created for CS and HA to allow for analysis of the patients that received a subsequent TKA, as differences in demographics were observed.

### 2.2. Outcome Variables

Time to total knee arthroplasty was the primary outcome measure for this study, defined as the time between the first instance of the respective IA injection (CS or HA) and the total knee arthroplasty in the same laterality. Survival analysis and comparison of the outcome measure were completed between HA and CS cohorts.

### 2.3. Demographic Variables

The database was queried to define demographics of each total injection cohort as well as the subgroups that underwent a subsequent TKA. These demographic variables included age, gender, and comorbidities. Additional variables in regard to the region and service location were included to further describe the populations.

### 2.4. Data Analyses

Continuous variables such as ages were compared using independent sample t-tests. Categorical variables, including certain demographics, comorbidities, and complications, were compared using Chi-square tests in bivariate analyses. A 4:1 case match was utilized to compare subsequent TKA groups with case match variables defined as age, Charlson comorbidity index (CCI), gender, alcohol abuse, diabetes, tobacco use, and obesity. The ratio of 4:1 was chosen to maximize the HA population for the comparison to CS. Kaplan–Meier survival analysis was conducted to analyze the TKA-free survival of patients within each group at 6 months, 1 year, 2, years, 3 years, and 4 years. This was reported as TKA-free proportions with 95% confidence intervals (CI). The log-rank test was performed for comparisons between cohorts. All analyses were performed using R Studio (Statistic Department of the University of Auckland, Auckland, NZ, USA) with significance defined as *p* < 0.05.

## 3. Results

### 3.1. Total Population

The initial query produced three distinct groups representing populations that had either had one or multiple injections of only CS, HA, or PRP. The PRP cohort had a total population of 3240 patients, of which 71 (2.2%) received a subsequent TKA. The CS cohort had a total population of 1,382,572, of which 81,271 (5.9%) received a subsequent TKA. The HA cohort had a total population of 164,000, of which 13,044 (8.0%) received a subsequent TKA (Table 1 and Table 2). Due to the low population within the PRP group, this group was excluded from population comparisons between groups, and is included for descriptive purposes only. Differences were observed between the CS and HA groups based on age, gender percentage, and comorbidities, as detailed in Table 1. Given the differences, a 4:1 case match was performed to permit comparison of the primary outcome measure.

### 3.2. Subsequent TKA Population (Case Match 4:1 CS vs. HA)

The mean time to TKA from first injection was similar between the HA and the CS group (377.8 vs. 370.0 days, respectively) (Table 2).

**Figure 1:**
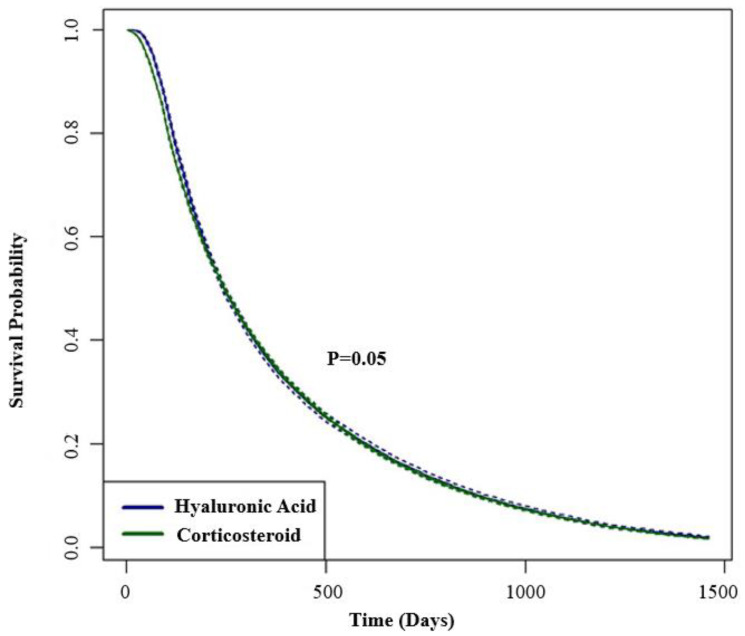
Kaplan–Meier curve; IA-Corticosteroid versus IA-HA (4:1 Case Match TKA Group).

The proportions of TKA-free survival for CS and HA at each timepoint, 6 months and annually until 4 years, are detailed in Table 3 and are similar between the two groups when survival is compared (*p* = 0.05) (Figure 1). 

## 4. Discussion

The analysis of a large, national healthcare database provides an opportunity to determine trends relating the use of various intra-articular injections and time until total knee arthroplasty. Understanding the time or the potential delay in TKA as a result of non-surgical therapies may be advantageous both from a clinical standpoint (optimalization of comorbidities prior to surgery, insurance implications, limiting treatment to one lifetime TKA) and from a healthcare cost perspective. A study reviewing a large database of ~2 million KOA concluded that utilization of IA-HA with conservative therapy for the first two years resulted in substantial cost savings by avoiding the primary and subsequent subset of revision TKA [9]. 

The results of this real-world study provide evidence that within a large cohort of KOA patients, mean time until TKA from first injection of either corticosteroids or hyaluronic acid is similar, at approximately 12 months. This evidence is contemporary given the downgrade in strength of recommendation within the latest American Academy of Orthopaedic Surgeons (AAOS) evidence-based clinical practice guideline for all three IA injections discussed in this study [10]. IA-HA has moderate strength of recommendation and is not recommended for routine use in the treatment of symptomatic KOA, and was downgraded due to lack of generalized research results. IA-CS also has moderate strength of recommendation as it could provide short-term relief for patients with symptomatic KOA. The downgrade in recommendation was due to the potential risk of accelerating OA, given recent published literature [8]. IA-PRP has a limited strength of recommendation, as it may reduce pain and improve function in symptomatic KOA, and was downgraded due to inconsistent evidence from published research. Estimates of IA-CS and HA use are higher than 50% of all patients with KOA, and regenerative medicine offering PRP has become an increasing practice for patients seeking proactive care.

The current study provides descriptive data of the population that was administered PRP for KOA; however, conclusions could not be formulated for the primary outcome measure due to the low population that received a subsequent TKA. Future research is needed once national databases have enough population to allow for meaningful analysis of trends. Stem cell treatment modalities such as injection of PRP are not an FDA-approved treatment for KOA and have been used increasingly [11,12,13,14,15] without clear evidence of benefit [16,17,18,19,20,21,22]. Overall utilization and offering of regenerative therapies have been expanding, with reports citing 570 clinics in 2015–2016, 715 in 2017–2018, and ~1000 in 2019 [23]. Although autologous stem cell therapies provide a higher level of comfort to patients as they utilize their own biologic material, there is limited evidence to support use of PRP as a disease-modifying treatment or as a method of delaying or preventing total knee arthroplasty (TKA). There is only one published retrospective study, of 186 patients, suggesting a delay in TKA of more than 1.5 years with a median delay of 5.3 years [24].

Despite significant differences between the total HA and CS cohorts found across multiple demographic variables, the differences are not clinically meaningful and are a result of the large difference in the total population size. The case match correction for comorbidities and demographics resolved the differences observed and provided two similar populations for comparison of the primary outcome measure. Prior published studies claim benefit of HA in delaying TKA [2,5,25,26] by assessing the time between diagnosis of KOA and TKA but utilized comparison groups of patients who did not receive HA rather than a direct comparison to another intra-articular injection. This is one of the strengths of the current study: the heterogeneity of comparator treatments is reduced, as well as the heterogeneity of the treatment timelines. Given the recent evidence that CS may contribute to accelerating KOA, the similarity of time to TKA between the HA and CS group in the current study raises concern regarding the actual efficacy of HA when assessed by a hard clinical endpoint (TKA).

There are multiple limitations to this study design using an administrative claims database. The study is limited to the data and variables collected, and not all relevant variables can be considered, such as KOA disease severity or grade at time of injection, which is critical for KOA treatment selection. Patients who had more than one type of injection were excluded and not analyzed in this study. Additionally, the time, volume, and transition of IA injection to another injection could not be clarified due to the limitations of the database. Socioeconomic factors of the patients may influence the time between the first injection and TKA but was not the focus of the study. The use of a matched design allowed us to mitigate some concerns, ensuring patient baseline comorbidities were nonfactors. Although PRP was not directly compared due to small patient numbers, baseline patient characteristics are demonstrated in Table 1. The PearlDiver database is primarily queried with ICD and CPT codes to create grouped data sets for comparison rather than granular inclusion and exclusion criteria. The three treatment groups were mutually exclusive but did not exclude or adjust for any other concurrent treatments. Lastly, since the database is limited in total time (six years), this study reports only those patients who had an injection and a TKA within this timespan; therefore, the actual time to TKA may be increased. The strength of our study lies in the large patient numbers obtained while utilizing a nationally representative sample.

Despite these limitations, the study design provides value given the large sample size and the real-world data on time between first knee intra-articular injection and total knee arthroplasty. With the downgrade in recommendations from AAOS on the use of all three IA injections for KOA, the use of these treatment options should be carefully considered in the era of accountable healthcare. The prevalent use of these treatments might not be warranted in every patient, and patients should be educated that these treatments will not contribute to the ultimate avoidance of the TKA. Expectations of delay to surgery or treatment benefit should be balanced with the cost and need for subsequent treatments (especially in the case of HA and PRP due to the cost burden). A prior large database study found that in the year prior to TKA, approximately 30% of the total non-inpatient cost was related to HA injections alone [27]. IA injections could be a prime target for reduction in cost in the treatment of KOA prior to TKA, and other less costly treatment options should be revisited with patients if planning the TKA is not desired.

## 5. Conclusions

Patients that received only IA-corticosteroids or IA-hyaluronic acid had a similar length of time between the first injection and the total knee arthroplasty associated with the injected joint. This evidence provides information for clinicians and patients alike when contemplating these non-surgical injection modalities for KOA. The similarity observed between these treatments supports the need for future research to determine whether there is any potential for reduction in healthcare costs for KOA treatment prior to TKA.

## Figures and Tables

**Table 1:** Baseline demographics by treatment group.

Variable	IA-PRP	IA-CS	IA-HA	*p*-Value *
Total N	3240	1,382,572	164,000	-
Subsequent TKA N (%)	71 (2.2)	81,271 (5.9)	13,044 (8.0)	-
Age Mean ± SD	63.0 ± 10.0	64.8 ± 11.5	64.8 ± 11.9	0.007932
Age Category N (%)				
18 to 19	18 (0.6)	210 (0.02)	67 (0.04)	<0.0001
20 to 24	40 (1.2)	1199 (0.1)	350 (0.2)	<0.0001
25 to 29	31 (1.0)	2950 (0.2)	611 (0.4)	<0.0001
30 to 34	54 (1.7)	7989 (0.6)	1381 (0.8)	<0.0001
35 to 39	116 (3.6)	18,805(1.4)	2829 (1.7)	<0.0001
40 to 44	174 (5.4)	39,006 (2.8)	4954(3.0)	<0.0001
45 to 49	275 (8.5)	71,724 (5.2)	8603 (5.2)	1.0
50 to 54	376 (11.6)	121,847 (8.8)	13,595 (8.3)	<0.0001
55 to 59	505 (15.6)	172,608 (12.5)	18,254 (11.1)	<0.0001
60 to 64	535 (16.5)	208,619 (15.1)	21,615 (13.2)	<0.0001
65 to 69	415 (12.8)	203,381 (14.7)	26,138 (15.9)	<0.0001
70 to 74	344 (10.6)	188,465 (13.6)	24,692 (15.1)	<0.0001
75 to 79	256 (7.9)	223,009 (16.1)	28,323 (17.3)	<0.0001
80 to 84	101 (3.1)	122,760 (8.9)	12,588 (7.7)	<0.0001
Male Gender N (%)	1485 (45.8)	500,034 (36.2)	62,669 (38.2)	<0.0001
Region N (%)				
Midwest	674 (20.8)	400,644 (30.0)	42,907 (26.2)	<0.0001
Northeast	682 (21.0)	294,311 (21.3)	40,686 (24.8)	<0.0001
South	1508 (46.5)	513,096 (37.1)	57,523 (35.1)	<0.0001
West	363 (11.2)	166,619 (12.1)	22,065 (13.5)	<0.0001
Unknown	13 (0.4)	7902 (0.6)	819 (0.5)	<0.0001
Service Location N (%)				
Clinic	0 (0)	12,959 (0.9)	499 (0.3)	<0.0001
Office	1213 (37.4)	1,213,283 (87.8)	145,371 (88.6)	<0.0001
Inpatient	495 (15.3)	165 (0.01)	0 (0)	0.0001
Other	0 (0)	1281 (0.09)	45 (0.03)	<0.0001
Outpatient	1519 (46.9)	148,276 (10.7)	17,024 (10.4)	<0.0001
Comorbidities N (%)				
Alcohol Use?	195 (6.0)	85,494 (6.2)	8202 (5.0)	<0.0001
Cancer	441 (13.6)	251,909 (18.2)	31,248 (19.1)	<0.0001
Coronary Artery Disease	725 (22.4)	414,040 (29.9)	48,522 (29.6)	0.002596
Chronic Kidney Disease	383 (11.8)	273,475 (19.8)	29,931 (18.3)	<0.0001
COPD	812 (25.1)	425,993 (30.8)	48,574 (29.6)	<0.0001
Congestive Heart Failure	134 (4.1)	98,897 (7.2)	11,193 (6.8)	<0.0001
Depression	1220 (37.7)	570,067 (41.2)	60,753 (37.0)	<0.0001
Diabetes	1074 (33.1)	596,587 (43.2)	70,136 (42.8)	0.002964
Diabetes Complicated	519 (16.0)	330,672 (23.9)	37,989 (23.2)	<0.0001
Diabetes Uncomplicated	880 (27.2)	486,913 (35.2)	57,285 (34.9)	0.02107
Hypertension	2056 (63.5)	1,097,128 (79.4)	125,741 (76.7)	<0.0001
Hypothyroidism	898 (27.7)	418,494 (30.3)	50,314 (30.7)	0.0006423
Liver Disease	544 (16.8)	263,193 (19.0)	29,546 (18.0)	<0.0001
Obesity	1488 (45.9)	693,586 (51.2)	76,982 (46.9)	<0.0001
Renal Disease	395 (12.2)	280,691 (20.3)	30,801 (18.8)	<0.0001
Renal Failure	395 (12.2)	280,409 (20.3)	30,766 (18.8)	<0.0001
Rheumatoid Arthritis	119 (3.7)	80,601 (5.8)	7651 (4.7)	<0.0001
Tobacco Use	1081 (33.4)	524,869 (38.0)	55,114 (33.6)	<0.0001

* Comparison of HA and CS groups only. Abbreviations. PRP = Platelet-Rich Plasma, CS = Corticosteroids, HA = Hyaluronic Acid, TKA = Total Knee Arthroplasty, N = Population, SD = Standard Deviation.

**Table 2:** Subsequent TKA, 4:1 Case Match Demographics (CS vs. HA).

Variable	IA-CS (4:1 Case Match)	IA-HA (4:1 Case Match)
Total N	45,124	11,492
Time to TKA Mean ± SD	370.0 ± 348.9	377.8 ± 349.2
Male Gender N (%)	16,645 (36.9)	4273 (37.2)
Age Mean ± SD	68.0 ± 8.6	67.9 ± 8.7
Comorbidities		
Alcohol Abuse	954 (2.1)	312 (2.7)
Cancer	8668 (19.2)	2283 (19.9)
Coronary Artery Disease	14,106 (31.3)	3583 (31.2)
Chronic Kidney Disease	8425 (18.7)	2099 (18.3)
COPD	13,246 (29.4)	3407 (29.6)
Congestive Heart Failure	2685 (6.0)	674 (5.9)
Depression	17,663 (39.1)	4412 (38.4)
Diabetes	19,226 (42.6)	4917 (42.8)
Diabetes Complicated	9829 (21.8)	2506 (21.8)
Diabetes Uncomplicated	15,316 (33.9)	3949 (34.4)
Hypertension	37,945 (84.1)	9527 (82.9)
Hypothyroidism	14,172 (31.4)	3697 (32.2)
Liver Disease	7425 (16.5)	1922 (16.7)
Obesity	24,241 (53.7)	6171 (53.7)
Renal Disease	9335 (20.7)	2157 (18.8)
Renal Failure	9337 (20.7)	2155 (18.8)
Rheumatoid Arthritis	2646 (5.9)	579 (5.0)
Tobacco Use	17,027 (37.7)	4383 (38.1)

**Table 3:** Cumulative proportions of TKA-free survival (Subsequent TKA Group; 4:1 Match).

	IA-CS (95% CI) N = 45,124	IA-HA (95% CI) N = 11,492
6 months	60.4 (59.9–60.8)	61.6 (60.7–62.5)
1 year	35.7 (35.3–36.2)	35.1 (34.2–35.9)
2 year	14.3 (14.0–14.7)	14.8 (14.2–15.5)
3 year	5.6 (5.4–5.9)	5.8 (5.4–6.3)
4 year	1.7 (1.6–1.8)	1.9 (1.7–2.2)

## Data Availability

Data can be given at the request of the authors as it is in our institution’s database.

## Appendix A

Knee Osteoarthritis: Right; ICD-10-D-M1711, ICD-10-D-M1731, ICD-10-D-M93261 or Left ICD-10-D-M1712, ICD-10-D-M1732, ICD-10-D-M93262

Platelet-Rich Plasma: CPT-0232T

Injections: CPT-20610, CPT-20611

Corticosteroids: CPT-J3300, CPT-J1030, CPT-J3301, CPT-J1040, CPT-J1700, CPT-J1710, CPT-J1720, CPT-C9256, CPT-J1094, CPT-J1020, CPT-J2920, CPT-J2930, CPT-J3302, CPT-J3303

Hyaluronic Acid: CPT-J7325, CPT-J7323, CPT-J7321, CPT-J7324, CPT-J7326, CPT-J7327, CPT-J7328, CPT-C9471, CPT-Q998
